# How Implicit Sequence Learning and Explicit Sequence Knowledge Are Expressed in a Serial Response Time Task

**DOI:** 10.5334/joc.439

**Published:** 2025-04-17

**Authors:** Marius Barth, Christoph Stahl, Hilde Haider

**Affiliations:** 1 Department of Psychology, University of Cologne, Germany

**Keywords:** implicit learning, sequence learning, drift-diffusion model

## Abstract

Sequence learning in the serial response time task (SRTT) is one of few learning phenomena where researchers agree that such learning may proceed in the absence of awareness, while it is also possible to explicitly learn a sequence of events. In the past few decades, research into sequence learning largely focused on the type of representation that may underlie implicit sequence learning, and whether or not two independent learning systems are necessary to explain qualitative differences between implicit and explicit learning. Using the drift-diffusion model, here we take a cognitive-processes perspective on sequence learning and investigate the cognitive operations that benefit from implicit and explicit sequence learning (e.g., stimulus detection and encoding, response selection, and response execution). To separate the processes involved in expressing implicit versus explicit knowledge, we manipulated explicit sequence knowledge independently of the opportunity to express such knowledge, and analyzed the resulting performance data with a drift-diffusion model to disentangle the contributions of these sub-processes. Results revealed that implicit sequence learning does not affect stimulus processing, but benefits response selection. Moreover, beyond response selection, response execution was affected. Explicit sequence knowledge did not change this pattern if participants worked on probabilistic materials, where it is difficult to anticipate the next response. However, if materials were deterministic, explicit knowledge enabled participants to switch from stimulus-based to plan-based action control, which was reflected in ample changes in the cognitive processes involved in performing the task. First implications for theories of sequence learning, and how the diffusion model may be helpful in future research, are dicussed.

Sequence learning in the serial response time task (SRTT) is one of few learning phenomena where researchers agree that such learning may proceed in the absence of awareness. In the classical paradigm (Nissen & Bullemer, 1987), stimuli are presented in several locations of a computer screen, and participants are instructed to press corresponding keys as quickly and accurately as possible. Unbeknownst to participants, stimuli are not presented in a random order, but follow an underlying sequential regularity. Over the course of learning, participants respond more quickly and accurately to stimuli that are presented in regular (compared with nonregular) stimulus locations. However, participants often fail to express explicit sequence knowledge in direct tests of such knowledge, such as verbal report, cued or free generation tasks, recognition tests, or process-dissociation measures (Buchner, Steffens, Erdfelder, & Rothkegel, 1997; Cohen, Ivry, & Keele, 1990; Curran & Keele, 1993; Destrebecqz & Cleeremans, 2001; Frensch & Rünger, 2003; Haider & Frensch, 2005; Willingham, Greeley, & Bardone, 1993; Willingham, Nissen, & Bullemer, 1989). It has therefore been concluded that, at least under specific circumstances, sequence learning may proceed in the absence of awareness.

There is no consensus on whether implicit and explicit learning are also based on two different learning mechanisms or systems, or whether both phenomena may be explained with a unitary learning mechanism. Cleeremans and Jiménez (2002) assume that implicit and explicit learning are based on a single learning system. Yet, the representations acquired through learning gradually differ in their strength, stability over time, and distinctiveness. Such *quality of representation* correlates with the accessibility of these representations to consciousness. Accordingly, the dissociation found between performance on the SRTT and measures of explicit sequence knowledge is explained by the fact that such measures are sensitive only to strong representations (that are also likely to be consciously accessible), whereas performance in the SRTT may also be driven by weaker representations that are less likely to be accessible to consciousness. Consciousness, however, allows to flexibly control the influence of acquired representations on behavior (i.e., the expression of learning).

The assumption of a unitary system is opposed to the view that two separate systems may mediate sequence learning. In their *dual-systems model*, Keele, Ivry, Mayr, Hazeltine, and Heuer (2003) distinguish between a *unidimensional* and a *multidimensional* learning system: In the multidimensional system, information from different feature dimensions can be processed together; the system depends on attention to protect it from overload. Processing in this system is initially automatic and unconscious; however, the information processed in the system is in principle accessible to consciousness. In contrast, processing in the unidimensional system takes place in a set of encapsulated, dimension-specific modules. This system operates independently of attention, because the encapsulation and separate processing of stimulus and response features already protects the system from overload; knowledge in this system is considered to be inaccessible to consciousness. Keele et al. (2003) themselves acknowledged that it is not clear what constitutes a dimension in their model, but maintain that (visual) features of the stimulus and (motor) features of the response constitute different dimensions.

## Representations and processes underlying implicit sequence learning

Abrahamse, Jiménez, Verwey, and Clegg (2010) proposed to view the dual-systems model, with its rich machinery of dimension-specific and dimension-independent processing, as a framework to integrate findings from earlier studies that investigated the representational basis of implicit sequence learning. In this line of research, it has been a long-standing debate whether implicit sequence learning is mediated by the formation of associations between consecutive stimuli, between consecutive responses, or between consecutive compounds of stimulus and response, where evidence for one type of representation has often been interpreted as evidence against another. Abrahamse et al. (2010) review a vast literature investigating the issue, arguing that response-based learning (i.e., associations between features of consecutive responses) and stimulus-based learning (i.e., associations between features of consecutive stimuli) received strong empirical support. Moreover, response-effect learning (i.e., associations between consecutive compounds of responses and subsequent stimuli, which may serve as action effects) received substantial support; Abrahamse et al. (2010) propose that this type of learning may proceed in the multidimensional system and, if sufficient attention is allocated, may become explicit. From the perspective of the dual-systems model, these different types of representations should not be considered as being mutually exclusive: Sequence representations may be based on different (combinations of) stimulus and response features that are available at different information-processing stages (Donders, 1969; Sternberg, 1969): Processing at the stimulus level (e.g., detection and encoding), processing at intermediate levels (e.g., response selection), and processing at the response level (e.g., response execution). Learning in the unidimensional system is, according to Abrahamse et al. (2010), confined to associating between consecutive instances of one particular type of stimulus feature or one type of response feature, while the multidimensional system may associate features from multiple dimensions, including combinations of stimulus and response features. Accordingly, response-effect learning may only proceed in the multidimensional system; by contrast, both response-based and stimulus-based learning may proceed in the unidimensional or in the multidimensional learning system.

From both the Keele et al. (2003) model and the Abrahamse et al. (2010) review, it is not completely clear which cognitive processes should be involved in the *expression* of these different types of learning. However, if unidimensional learning of a sequence of stimulus features is truly encapsulated within a dimension-specific processing module, it should selectively influence stimulus processing (i.e., detection and encoding); if unidimensional learning of a sequence of response features is truly encapsulated, it should selectively influence response processing (i.e., response execution). Response selection, on the other hand, should be unaffected by unidimensional learning, but may be affected by multidimensional learning (such as response-effect learning).

In contrast to the view of Abrahamse et al. (2010), an opposing view has been championed by E. H. Schumacher and colleagues, who argue that response selection is the locus of (implicit) sequence learning. E. H. Schumacher and Hazeltine (2016) summarized their response-selection account as positing that not simple associations, but hierarchically organized representations containing features of stimuli and responses, task goals, and drives are acquired in sequence learning that are acquired and expressed at the response-selection stage. Response selection is, however, typically considered to be a central process (Hazeltine & Schumacher, 2016), to be dependent on selective attention (i.e., the task set), and necessitates information about both stimuli and responses. While this view is compatible with a unitary learning system as proposed by Cleeremans and Jiménez (2002), it poses a challenge to the dual-systems model, because these features are considered to be characteristic of only the multidimensional learning system. If implicit sequence learning indeed relied on response-selection learning, this would render the unidimensional learning system superfluous.

Important to the question whether an involvement of response selection in implicit sequence learning is compatible with the assumptions of the dual-systems model, it should be noted that Haider and colleagues propose an alternative view on the dual-systems model that is compatible with an involvement of response selection in implicit sequence learning. They propose that the unidimensional learning system may not be separated along features of stimuli and responses, but along *abstract feature dimensions* such as spatial location, shape, or color (Eberhardt, Esser, & Haider, 2017; Haider, Esser, & Eberhardt, 2020). If, indeed, the unidimensional learning system included a processing module specific to spatial location, stimulus and response locations could be represented together within such a module, providing the necessary information to subserve learning at the response-selection stage. Such a view would also explain findings that suggest that, irrespective of the modality (stimulus or response), spatial sequences are much easier to learn than symbolic sequences (e.g., Koch & Hoffmann, 2000).

## Explicit sequence knowledge

Previous research indicates that explicit sequence knowledge affects SRTT performance in a different way than implicit sequence knowledge. The emergence of explicit sequence knowledge in the SRTT is frequently accompanied with sudden decreases of response times (*RT drops*) during training (Haider & Frensch, 2005; Haider & Rose, 2007), and it has been an open question whether such RT drops are a precursor of sequence awareness (i.e., an expression of implicit sequence learning) or a consequence of participants first acquiring explicit sequence knowledge and then switching response strategies (i.e., an expression of explicit sequence knowledge). The *Unexpected Event Hypothesis* (Frensch et al., 2003; for a recent discussion, see Esser, Lustig, & Haider, 2021) postulates that experiencing an unexpected event triggers a search process that may result in the discovery of the sequential regularity. In line with such reasoning, multiple studies (Esser & Haider, 2017; Rünger & Frensch, 2008; Schwager, Rünger, Gaschler, & Frensch, 2012) found that inserting unexpected events into the SRTT indeed resulted in more explicit sequence knowledge. Moreover, using a combination of EEG and fMRI studies, Rose, Haider, and Büchel (2010) and Wessel, Haider, and Rose (2012) found that RT drops were accompanied by increases in neural coupling between distant brain areas and an increase in neural activity in brain areas that have been discussed as being involved in the processing of predictions and prediction errors. They interpreted these findings as evidence in favor of an increase in error processing that may serve as an unexpected event that triggers the emergence of sequence awareness. Other authors, however, reasoned that participants first acquire explicit sequence knowledge, and then switch response strategies. Tubau, Hommel, and López-Moliner (2007) hypothesized that participants switch from *stimulus-based* to *plan-based* action control, where the stimulus-based control mode represents an efficient strategy to delegate control to external information (i.e., the imperative feature of the stimulus). By contrast, the plan-based control mode refers to the construction of an action plan that consists of an ordered sequence of action effects. Koch (2007) argued that explicit sequence learning may lead to motor chunking, where planned sequences of responses are executed independent of (potentially conflicting) stimulus information. Such reasoning is supported by studies that found that effects of response-selection conflict or difficulty were reduced if participants acquired explicit sequence knowledge: Hoffmann and Koch (1997) found that RT differences between easy and difficult stimuli disappeared for explicit learners, Haider, Eichler, and Lange (2011) demonstrated that participants who showed an RT drop (i.e., explicit learners) also showed reduced Stroop-congruency effects. Tubau et al. (2007) found reduced response-repetition costs only for explicit learners, Koch (2007) found that participants who acquired explicit sequence knowledge showed reduced Simon effects.

Recently, Lustig, Esser, and Haider (2022) directly tested whether RT drops in the SRTT are either a precursor of explicit sequence knowledge or a consequence of a switch to plan-based action control. To this end, they manipulated the ease of producing RT drops in the SRTT by manipulating the RSI in either a predictable or a random fashion, assuming that if RT drops are a precursor of explicit knowledge, hampering such drops should reduce the acquisition of explicit sequence knowledge. If, instead, RT drops are indicative of a strategy shift from stimulus-based to plan-based action control that is possible as soon as explicit sequence knowledge has been acquired and the task design (and materials) allow to use this knowledge. Lustig et al. (2022) found that RT drops are not a precursor of explicit conscious insight, but rather a consequence of a switch to plan-based action control. Yet, it remains an open question how a switch to plan-based action control may be reflected in the cognitive processes involved in the expression of sequence knowledge.

## Separating the processes involved in the expression of sequence knowledge

To investigate which cognitive processes are involved in the expression of implicit and explicit sequence knowledge, we here propose to apply the drift-diffusion model to the SRTT. The DDM has originally been proposed as a theory of memory retrieval (Ratcliff, 1978), but has been successfully applied to many speeded-choice tasks; specifically, it maps changes in response times and error rates onto parameters quantifying psychologically meaningful cognitive processes (for reviews, see Ratcliff, Smith, Brown, & McKoon, 2016; Voss, Nagler, & Lerche, 2013; Wagenmakers, 2009). We will first provide an overview of the DDM and how it can be applied to SRTT performance data; then, we will briefly summarize the results from an earlier reanalysis of two SRTT experiments using the DDM.

**Figure 1:**
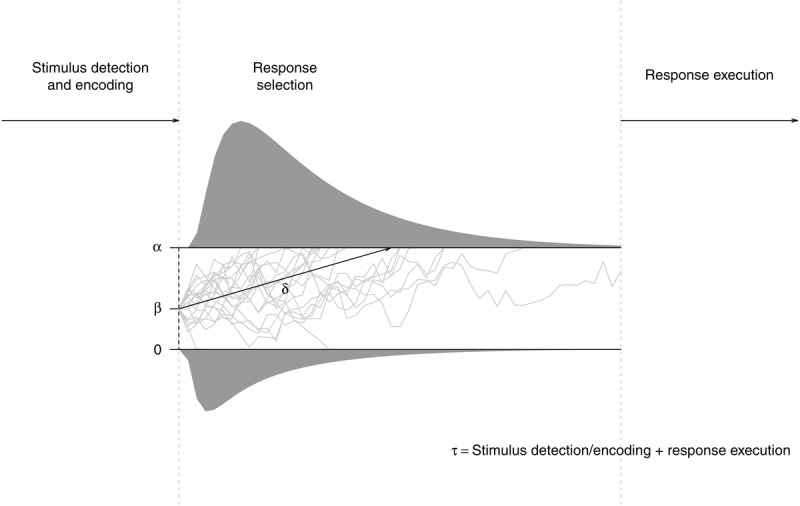
The diffusion model. On each trial, the decision process (depicted as grey lines) begins at a starting point that is determined by parameter β. The spread of the thresholds is determined by parameter α. Evidence is accumulated in a random-walk fashion. When one of the two thresholds is reached, a decision is made. The average rate of evidence accumulation is determined by parameter δ. The decision process is preceded by stimulus encoding and succeeded by response execution, the duration of both processes is captured by nondecision time τ. This basic diffusion model may be extended by response-competition parameter ξ and inter-trial variabilities for the model’s core parameters.

Figure 1 illustrates the processing assumptions of the DDM. It is assumed that on each trial, evidence is accumulated in a noisy fashion to reach a decision about the to-be-selected response. The average rate of evidence accumulation (drift rate δ) is dependent on the quality of information from the stimulus or a match with memory. In the SRTT, harder-to-discriminate stimuli or more difficult S–R mappings should affect this parameter; moreover, if indeed consecutive S–R rules are learned in the SRTT, this would result in a memory match for regular responses and a mismatch for nonregular responses. The starting point of the decision process (parameter β) captures information that is already available before the stimulus is presented. In the SRTT, it may capture an anticipation of the to-be-selected response, which may be driven by either R–R or S–R associations. The spread between both thresholds captures the decision criterion (i.e., response caution α). Extradecisional processes, such as stimulus detection, its encoding, and response execution are captured by nondecision time τ. If stimulus-based learning (i.e., the formation of S–S associations) contributes to implicit sequence learning, nondecision time should be affected by stimulus regularity. It is also possible to further disentangle stimulus processing and response-related differences in nondecision time for regular vs. nonregular responses by estimating an additional response-competition parameter ξ (Voss, Rothermund, Gast, & Wentura, 2013; Voss, Voss, & Klauer, 2010); purely-motor learning (effector-specific or not) could selectively mediate such a response-competition effect. The *full* DDM also assumes inter-trial variability in starting point, drift rate, and nondecision time; moreover, most recent advances in DDM modelling allow to capture temporal changes in parameter estimates (for an overview, see L. Schumacher, Schnuerch, Voss, & Radev, 2024), and there are first examples of how such models can be used to investigate learning curves (Cochrane, Sims, Bejjanki, Green, & Bavelier, 2023).

In a first study, we reanalyzed two SRTT experiments with the DDM (details can be found in Barth, 2018). In Experiment 1, participants worked on probabilistic materials with 60% regular trials (i.e., with a probability of .6, the next stimulus followed the sequential structure; with a probability of .4, another stimulus that violated the sequence was presented). Such materials are typically considered to enable robust sequence learning while participants remain largely unaware of the sequence (Barth, Stahl, & Haider, 2019; Jiménez & Méndez, 1999). By contrast, in Experiment 2, stimulus materials consisted of chunks of 15 to 22 stimulus locations that followed a deterministic six-item first-order conditional sequence interrupted with chunks of random trials of the same length. With these materials, participants were well able to acquire substantial amounts of explicit sequence knowledge.

A comparison of both experiments revealed qualitatively different patterns of effects on diffusion model parameters. In Experiment 1, (implicit) sequence learning was reflected in changes in drift rate and response competion, whereas in Experiment 2, (explicit) sequence learning was reflected in changes in starting point. An involvement of response-selection processes (drift rate) in the expression of implicit sequence learning suggests that the representations acquired in implicit sequence learning may contain information about both stimuli and responses, while response-competition effects may reflect additional, response-based learning. The finding that explicit sequence knowledge was reflected in changes in starting point suggests that explicit sequence knowledge be mediated by other cognitive processes, and that these rely on qualitatively different representations that may be acquired in the SRTT. Yet, the results of this study are limited by the fact that the opportunity to gain explicit knowledge was confounded with the ability to switch response strategy: Probabilistic materials did not allow for reliably predicting the next stimulus, and therefore encouraged stimulus-based action control. By contrast, materials that contain deterministic chunks might encourage a switch to plan-based action control if participants are able to detect differences between regular and deterministic chunks (for instance, by detecting changes in fluency). It is therefore well conceivable that effects on different model parameters do not reflect differences in representation, but indicate a switch from stimulus-based to plan-based action control. Moreover, the results of this study are further limited by the fact that we did not use formal model comparisons to test for effects on the models’ parameters. In conjunction with the low error rates in both experiments, and the known issue that parameter identifiability suffers under such circumstances (Lüken, Heathcote, Haaf, & Matzke, 2025), it might be that we did not find learning effects on some parameters because of a lack of statistical power. The results of this reanalysis should therefore be considered preliminary.

## Aims of the present study

In the present study, we aim to separate the processes involved in the expression of implicit and explicit sequence knowledge. To this end, participants perform a standard SRTT with a sequence of stimulus locations and (corresponding, spatially compatible) motor responses. We manipulate explicit sequence knowledge by revealing, to a subset of participants, the sequence of stimulus locations (and, hence, motor responses). Orthogonally, we manipulate the possibility to switch to plan-based action control by using either probabilistic or mixed-deterministic stimulus materials.

Participants who work on probabilistic materials and do not receive advance knowledge of the sequence may be considered our baseline condition: Such materials have been found to generate substantial sequence learning while participants remain largely implicit about the sequence (Barth et al., 2019; Jiménez & Méndez, 1999). In this group, it should therefore be possible to explore the processes involved in the expression of implicit sequence learning. Comparing this group with participants who work on probabilistic materials but also receive advance knowledge of the sequence, it is possible to explore whether explicit knowledge *per se* is expressed differently than implicit sequence learning. Comparing probabilistic and deterministic materials in fully explicit participants, it is possible to explore which processes are affected by switching to plan-based (versus stimulus-based) action control. Finally, participants who did not receive advance knowledge of the sequence but worked on deterministic materials complete the picture: If deterministic materials indeed facilitate the acquisition of explicit sequence knowledge, these participants should also be able to switch to plan-based action control as soon as they acquired explicit sequence knowledge. However, it might be the case that acquired explicit sequence knowledge is expressed differently than explicit sequence learning that has been revealed to participants in advance.

## Method

### Participants

One hundred and twenty-three participants (104 women) aged between 18 and 49 years (*Md* = 23 years) completed the study. Most were undergraduates from University of Cologne. Participants were randomly assigned to experimental conditions. They received either course credit or 7.50 Euro for their participation.

### Materials and Procedure

Participants worked on an SRTT consisting of 14 blocks with 144 trials each (for a total of 2,016 responses), the experimental software was written and run with *PsychoPy* (Version 2023.2.1, Peirce et al., 2019). The experiment was run on 24” LCD monitors with a screen resolution of 1,920 × 1,080px. The viewing distance was approximately 60 cm. A horizontal sequence of six black squares (72px each) with mid-gray outlines were presented on a black screen. The distance between squares was 72px. Each screen location corresponded to one of six keys on a QWERTZ keyboard (from left to right Y, X, C, comma, period, minus), which were marked with red stickers. Participants were instructed to place their ring, middle, and index fingers of both hands on these keys. They were instructed to press the corresponding key whenever a square’s color changed from black to mid-gray. On each trial of the SRTT, after a response-stimulus interval (RSI) of 250 ms, the imperative stimulus was presented until a response was given. If a response was given before the stimulus had occurred, for 2 sec, participants were reminded to press a key only after the stimulus had occurred. To obtain a significant proportion of error responses, we used a response deadline of 500 msec. To allow participants to get used to the task, in the first block, we used response deadlines of 900, 800, 700, 600 msec for 24 trials each before switching to 500 msec for the remainder of the experiment. If the response deadline had been exceeded, a warning sign together with a reminder to answer more quickly, even at the expense of committing more errors, was presented for 1200 msec. If the wrong key had been pressed, “wrong key” was presented for 300 msec.

For each participant anew, we generated a random permutation of the six possible stimulus locations which served as the six-item sequence. For participants in the *probabilistic sequence* conditions, stimulus locations followed this sequence with a probability of .6; otherwise, another stimulus location was randomly selected from a uniform distribution (excluding immediate repetitions). For participants in the *mixed-deterministic sequence* conditions, stimulus locations deterministically followed this six-item sequence in deterministic blocks; in random blocks, stimulus locations were randomly selected from a uniform distribution (excluding immediate repetitions). The order of random vs. deterministic blocks was randomly selected for these participants: Either blocks 1, 3, 5, 7, 9, 11, 13, or 2, 4, 6, 8, 10, 12, 14 were deterministic. Note that in random blocks, approximately 1/5 = 20% of stimuli followed the sequence by chance. Hence, all participants received stimulus materials that consisted of approximately 60% regular stimulus locations.

Prior to the SRTT, participants in the *sequence concealed* conditions did not receive any a-priori information that stimulus locations would follow an underlying sequential structure. By contrast, participants in the *sequence revealed* conditions were informed that stimulus locations followed such a regularity. If these participants worked on probabilistic materials, they were informed that on 60% of trials, the stimuli would be presented in a regular location, and in 40% of trials, they would be randomly selected. If these participants worked on mixed-deterministic materials, they were informed about the blocked structure of deterministic vs. random blocks, with exact information about in which blocks stimuli would follow the sequence and in which blocks stimulus locations would be randomly selected. Moreover, participants in the *sequence revealed* conditions were explicitly encouraged to try to use their advance sequence knowledge to optimize task performance. They were then informed about the exact six-item structure of their sequence (e.g., “2–1–6–5–3–4”) and they performed 18 trials of the sequence (i.e. three repetitions of the full sequence).

Following the SRTT, we assessed explicit sequence knowledge in a post-experimental interview. Participants were told that they had been assigned to an experimental condition with or without a sequential structure, and were asked to indicate if they believed that they were in a sequenced or a random condition. If they believed that they were in a sequenced condition, they were then asked to freely reproduce the sequence. Finally, all participants were asked to reproduce the sequence in a forced-choice manner. Participants were then thanked and debriefed.

### Design

Between participants, we manipulated *material* (probabilistic vs. mixed deterministic) and *instructions* (sequence concealed vs. sequence revealed). Each participant worked on 14 SRTT blocks that were collapsed into seven *block pair*s.

Within probabilistic materials, two trial types may be distinguished: *Nonregular* trials that did not follow the six-item sequence, and *regular* trials that did follow the sequence. With mixed-deterministic materials, each block pair was composed of a random block (where stimuli were randomly selected) and a deterministic block (where stimuli always followed the sequence). Hence, three trial types may be distinguished: *Nonregular* trials and *regular* trials from random blocks (remember that single transitions could follow the sequence by chance), and *deterministic* trials from deterministic blocks.

### Data preparation

Because an SRTT with a response deadline can be a demanding task, we screened participant data for the proportion of too-slow responses and error rates: We excluded participants who, in one of block pairs 2–7, exceeded an error rate of 50% or responded too slowly on more than 30% of trials. We also excluded data of two participants who did not finish the study and of five participants whose response times were not properly saved. After applying these exclusion criteria, 25 participants remained in the mixed deterministic, sequence concealed group; 29 participants remained in the probabilistic, sequence concealed group; 26 participants remained in the mixed deterministic, sequence revealed group; and 26 participants remained in the probabilistic, sequence revealed group.

Trials that followed an erroneous response or a response that exceeded the response deadline (i.e., post-feedback trials) were excluded from analyses. We also excluded the first four trials of each block, and trials with responses faster than 20 ms or slower than 2 s.

## Results

Before applying the drift-diffusion model, we first analyzed response times and error rates from the SRTT and the responses from the post-experimental interview with linear models.^1^

### Response times and error rates

For ease of interpretation, we report three separate analyses for both response times and errors: One for the data from the probabilistic groups, and one each for the deterministic and random blocks from the mixed-deterministic groups. Subsequently, we report analyses of two distinct types of error responses: Errors that follow the regularity of motor responses, and errors that do not follow that regularity.

#### Probabilistic materials

**Figure 2:**
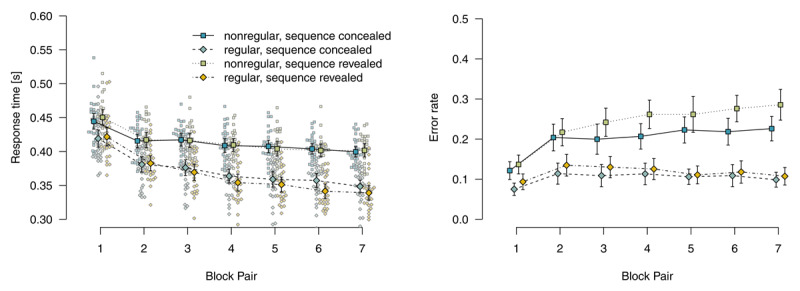
Mean response times for correct responses and error rates in probabilistic materials. Error bars represent 95% (between-subjects) confidence intervals, small points represent individual participants’ means.

Figure 2 shows mean response times for correct responses and error rates for the probabilistic materials. Analyzing response times for correct responses using a 2 (*instructions*: sequence concealed vs. revealed) × 2 (*stimulus-location regularity*: regular vs. nonregular) × 7 (*block pair*) ANOVA, we found a main effect of *block pair, F*(2.95,306.84) = 267.55, *p* < .001, \[
\hat{\eta}_{G}^{2}\] = .148, reflecting decreasing response times over blocks. We also found a main effect of *stimulus-location regularity, F*(1,104) = 194.80, *p* < .001, \[
\hat{\eta}_{G}^{2}\] = .423, and a significant two-way interaction of *block pair* and *stimulus-location regularity, F*(2.79,290.40) = 59.80, *p* < .001, \[
\hat{\eta}_{G}^{2}\] = .028: Responses were increasingly faster for regular compared with nonregular trials, indicating sequence-specific learning. Responses in regular trials were descriptively faster in the sequence-revealed (compared with the sequence-concealed) condition, but all remaining model terms were not significant, all other *p*s ≥ .135.

An analogous ANOVA of error rates revealed a main effect of *block pair, F*(4.63,245.16) = 34.32, *p* < .001, \[
\hat{\eta}_{G}^{2}\] = .099, a main effect of *stimulus-location regularity, F*(1,53) = 277.26, *p* < .001, \[
\hat{\eta}_{G}^{2}\] = .359, and a two-way interaction of *block pair* with *stimulus-location regularity, F*(5.17,274.03) = 22.39, *p* < .001, \[
\hat{\eta}_{G}^{2}\] = .050: Error rates were increasingly higher for nonregular compared with regular trials, also indicating sequence learning. Neither the main effect of *instructions, F*(1,53) = 2.98, *p* = .090, \[
\hat{\eta}_{G}^{2}\] = .033, nor the interaction of *instructions* with *block pair* was significant, *F*(4.63,245.16) = 0.80, *p* = .544, \[
\hat{\eta}_{G}^{2}\] = .003. These above effects were qualified by a significant three-way interaction, *F*(5.17,274.03) = 2.48, *p* = .031, \[
\hat{\eta}_{G}^{2}\] = .006 and a two-way interaction of *instructions* with *stimulus-location regularity, F*(1,53) = 4.06, *p* = .049, \[
\hat{\eta}_{G}^{2}\] = .008. To disentangle these interactions, we analyzed regular and nonregular trials, separately. Analyzing only regular trials, we found only a main effect of *block pair, F*(4.69,248.42) = 9.08, *p* < .001, \[
\hat{\eta}_{G}^{2}\] = .041, reflecting increasing error rates over blocks, all other *p*s ≥ .348. By contrast, analyzing only nonregular trials, we found a main effect of *block pair, F*(5.22,276.40) = 37.58, *p* < .001, \[
\hat{\eta}_{G}^{2}\] = .183, and a main effect of *instructions, F*(1,53) = 4.41, *p* = .040, \[
\hat{\eta}_{G}^{2}\] = .054, with more errors in the sequence-revealed condition. The interaction of *block pair* and *instructions*, reflecting the descriptive observation that the difference between conditions increased over block pairs, was not significant, *F*(5.22,276.40) = 1.91, *p* = .090, \[
\hat{\eta}_{G}^{2}\] = .011.

To summarize, for participants who worked on probabilistic materials, we found robust sequence learning in both response times and error rates. Revealing the sequence to such participants had only small effects on response times: Responses were only descriptively faster for regular trials. However, error rates were higher for nonregular trials. We interpret this finding as first evidence that participants in the sequence-revealed condition actually tried to use their explicit sequence knowledge, but were hard-pressed to do so. In the probabilistic material group, top-down attempts to apply explicit knowledge appear to have interfered with performance (instead of facilitating it). Interference is suggested by the specificity of the finding; in an alternative account in terms of a more liberal decision criterion (e.g., by frustrating participants with a virtually impossible task), response times for nonregular trials and error rates for regular trials should also have been affected. More research is needed to better pin down such a possible interference and to determine its boundary conditions.

#### Deterministic blocks of mixed-deterministic materials

**Figure 3:**
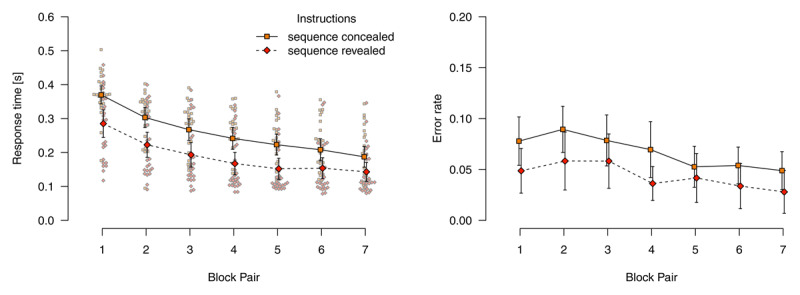
Response times for correct responses and error rates in deterministic blocks. Error bars represent 95% (between-subjects) confidence intervals, small points represent individual participants’ means.

Figure 3 shows mean response times for correct responses and error rates in deterministic blocks. Analyzing response times for correct responses using a 2 (*instructions*: sequence concealed vs. revealed) × 7 (*block pair*) ANOVA, we found a main effect of *block pair, F*(2.79,136.47) = 128.09, *p* < .001, \[
\hat{\eta}_{G}^{2}\] = .312, reflecting decreasing response times over blocks. We also found a main effect of *instructions, F*(1,49) = 11.64, *p* = .001, \[
\hat{\eta}_{G}^{2}\] = .164, response times were faster in the *sequence-revealed* condition. Although, descriptively, the RT difference between sequence concealed compared with sequence revealed conditions decreased, these main effects were not qualified by an interaction, *F*(2.79,136.47) = 2.10, *p* = .108, \[
\hat{\eta}_{G}^{2}\] = .007.

An analogous ANOVA for error rates revealed only a main effect of *block pair, F*(4.31,210.95) = 10.21, *p* < .001, \[
\hat{\eta}_{G}^{2}\] = .048, reflecting decreasing error rates over blocks. Neither the main effect of *instructions, F*(1,49) = 3.01, *p* = .089, \[
\hat{\eta}_{G}^{2}\] =.044. nor the interaction were significant, *F*(4.31,210.95) = 0.88, *p* = .481, \[
\hat{\eta}_{G}^{2}\] = .004.

We conclude that, in deterministic blocks, participants were well able to use their explicit knowledge to improve task performance. Although participants in the sequence concealed condition acquired substantial amounts of sequence knowledge (likely explicit knowledge; see the results of the post-experimental interview reported below), the speed with which they performed the task did not reach that of participants in the sequence-revealed condition (while both reached comparable levels of accuracy).

#### Random blocks of mixed-deterministic materials

**Figure 4:**
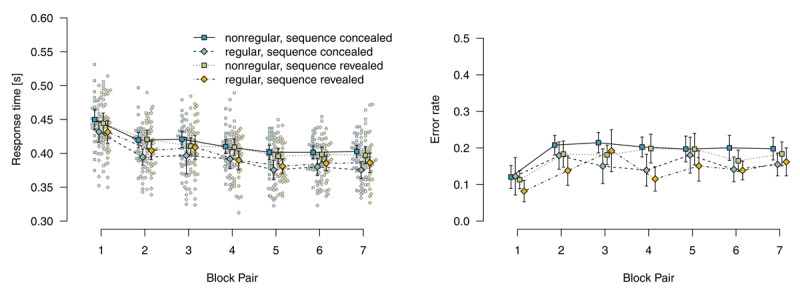
Response times for correct responses and error rates in random blocks. Error bars represent 95% (between-subjects) confidence intervals, small points represent individual participants’ means.

Figure 4 shows mean response times for correct responses and error rates in random blocks. Note that, in random blocks, it is also possible to distinguish between nonregular and regular stimulus locations because in a fifth of trials, stimulus locations adhere to the sequence by chance. Analyzing response times for correct responses using a 2 (*instructions*: sequence concealed vs. revealed) × 2 (*stimulus-location regularity*: regular vs. nonregular) × 7 (*block pair*) ANOVA, we found a main effect of *block pair, F*(3.78,185.09) = 49.70, *p* < .001, \[
\hat{\eta}_{G}^{2}\] = .204, response times decreased over blocks. We also found a main effect of *stimulus-location regularity, F*(1,49) = 59.77, *p* < .001, \[
\hat{\eta}_{G}^{2}\] = .069, with faster responses for regular (vs. nonregular) stimulus locations. This effect of regularity indicates that sequence learning was expressed in these blocks. The interaction of *block pair* and *stimulus-location regularity* was not significant, *F*(4.69,230.03) = 0.55, *p* = .727, \[
\hat{\eta}_{G}^{2}\] = .002. The main effect of *instructions* was not significant, *F*(1,49) = 0.01, *p* = .905, \[
\hat{\eta}_{G}^{2}\] = .000. *Instructions* entered into an interaction with *stimulus-location regularity, F*(1,49) = 4.86, *p* = .032, \[
\hat{\eta}_{G}^{2}\] = .006: The RT advantage for regular stimulus locations was bigger if the sequence had been revealed. All other *p*s ≥ .420.

Mirroring RT results, a parallel ANOVA for error rates revealed main effects of *block pair, F*(4.67,228.97) = 10.88, *p* < .001, \[
\hat{\eta}_{G}^{2}\] = .068, and *stimulus-location regularity, F*(1,49) = 29.39, *p* < .001, \[
\hat{\eta}_{G}^{2}\] = .042. The interaction of *block pair* and *stimulus-location regularity* was not significant, *F*(5.18,253.76) = 1.72, *p* = .128, \[
\hat{\eta}_{G}^{2}\] = .009. All model terms including *instructions* were not significant, all *p*s ≥ .164.

To summarize, participants who worked on mixed-deterministic materials expressed sequence learning not only in deterministic blocks (where it is possible to process the task in a plan-based fashion), but also in the random blocks (where only stimulus-based action control is adaptive).

### More errors through sequence learning?

In the probabilistic and random (but not deterministic) materials, a stimulus was sometimes presented in a nonregular stimulus location and thus prompted a nonregular response. In this case, two distinct types of error responses may be distinguished: Error responses that follow the regularity of motor responses (i.e., rule-adhering errors), and error responses that do not follow that regularity. If, over the course of learning, participants tend to generate rule-adhering errors more frequently, this would indicate that response-selection processes (i.e., processes that may impact *which* response is selected) are involved in sequence learning. To test this notion, we analyzed error responses (excluding post-error and post-feedback trials as well as response repetitions) and calculated the proportion of rule-adhering errors.

Analyzing these proportions with an ANOVA revealed a main effect of *material, F*(1,101) = 7.16, *p* = .009, \[
\hat{\eta}_{G}^{2}\] = .025, with more rule-adhering errors in probabilistic materials, all other *p*s ≥ .125. We also tested these proportions against chance baseline (i.e., 1/4 = .25, given that response repetitions and correct responses are excluded from this analysis). The proportion of rule-adhering errors exceeded the chance baseline in both conditions: For random blocks of mixed-deterministic material, it was *M* = 0.28, 95% CI [0.26, ∞], *t*(101) = 2.21, *p* = .015. For probabilistic materials, it was *M* = 0.33, 95% CI [0.31, ∞], *t*(101) = 6.19, *p* < .001. This trend toward rule-adhering errors suggests that response selection may have been impacted by sequence learning. The effect was stronger for participants who worked on probabilistic materials, which might be explained with differences in processing mode, or with different representations underlying sequence-specific effects in these conditions.

### Faster errors through sequence learning?

**Figure 5:**
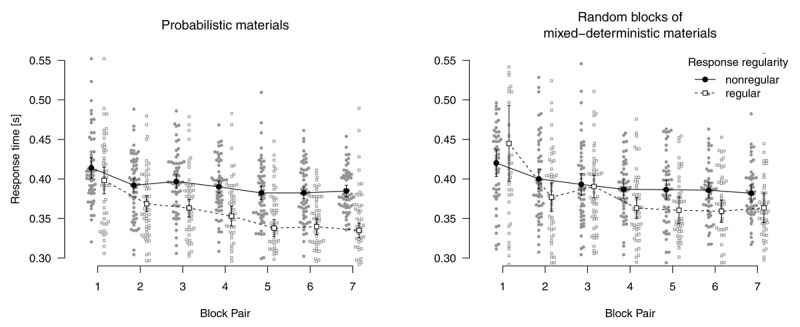
Response times for error responses. Error bars represent 95% within-subjects confidence intervals, small points represent individual participants’ means.

We also analyzed response times (see Figure 5) for error responses (again excluding post-error trials) and found a main effect of *response regularity, F*(1,76) = 60.49, *p* < .001, \[
\hat{\eta}_{G}^{2}\] = .049, a main effect of *block pair, F*(3.14,238.48) = 25.97, *p* < .001, \[
\hat{\eta}_{G}^{2}\] = .106, and their interaction, *F*(2.85,216.53) = 4.68, *p* = .004, \[
\hat{\eta}_{G}^{2}\] = .016. Moreover, the interactions of *material* and *response regularity, F*(1,76) = 15.45, *p* < .001, \[
\hat{\eta}_{G}^{2}\] = .013, and of *block pair* and *response regularity* were significant, *F*(2.85,216.53) = 4.68, *p* = .004, \[
\hat{\eta}_{G}^{2}\] = .016, all other *p*s ≥ .144. To disentangle these interactions, we analyzed probabilistic and random materials separately (note that the deterministic material contained no relevant trials): For probabilistic materials, we found a main effect of *block pair, F*(4.09,180.10) = 20.39, *p* < .001, \[
\hat{\eta}_{G}^{2}\] = .126, a main effect of *response regularity, F*(1,44) = 99.80, *p* < .001, \[
\hat{\eta}_{G}^{2}\] = .140, and their interaction, *F*(4.21,185.35) = 5.40, *p* < .001, \[
\hat{\eta}_{G}^{2}\] = .026, all other other *p*s ≥ .135. Participants not only produced an above-chance proportion of rule-adhering errors, but these error responses also became increasingly faster over blocks. For random materials, we only found a main effect of *block pair, F*(2.41,74.62) = 8.93, *p* < .001, \[
\hat{\eta}_{G}^{2}\] = .096, reflecting decreasing response times over blocks, and a main effect of *response regularity, F*(1,31) = 4.96, *p* = .033, \[
\hat{\eta}_{G}^{2}\] = .010. all other *p*s ≥ .274, In the random blocks, responses were also faster for rule-adhering (or motor-regular) responses.

In sum, the effect of motor regularity on response times mirrored the result that rule-adhering errors are chosen above chance. In other words, when stimuli were presented in nonregular locations and a wrong key was chosen, participants were not only more likely to select a regular response, but were also faster when doing so. It is not clear whether such an effect may be attributable to changes in response tendencies or response-selection learning, or whether this is a motor-specific effect attributable to extensive practice of the same consecutive responses.

### Post-experimental interview

Participants were asked whether they believed they had been working on materials containing a sequence (and to name that sequence); and then were presented with each stimulus and asked to select the next one in the sequence (i.e., forced choice). We first analyzed how many participants indicated that they believed that they were in a sequenced condition. For mixed-deterministic materials, 25 out of 26 participants in the sequence-revealed condition indicated that they were in a sequenced condition; 20 out of 23 participants in the sequence-concealed condition indicated the same. For participants who worked on probabilistic materials, only 12 out of 26 participants in the sequence-revealed condition and 14 out of 27 participants in the sequence-concealed condition believed that they had worked on sequenced materials.

Regarding free-recall performance, we analyzed the number of correctly generated transitions using a 2 (*material*: probabilistic vs. mixed deterministic) × 2 (*instructions*: sequence concealed vs. sequence revealed) ANOVA that revealed a main effect of *material, F*(1,98) = 27.60, *p* < .001, \[
\hat{\eta}_{G}^{2}\] = .220, a main effect of *instructions, F*(1,98) = 5.51, *p* = .021, \[
\hat{\eta}_{G}^{2}\] = .053, and no interaction, *F*(1,98) = 0.08, *p* = .779, \[
\hat{\eta}_{G}^{2}\] = .001. Participants who worked on mixed-deterministic materials recalled more transitions than those who worked on probabilistic materials. Also, revealing the sequence to participants resulted in more correctly recalled transitions.

Regarding forced-choice performance, we analyzed the proportion of correct responses using a 2 (*material*: probabilistic vs. mixed deterministic) × 2 (*instructions*: sequence concealed vs. sequence revealed) ANOVA that revealed a main effect of *material, F*(1,98) = 25.50, *p* < .001, \[
\hat{\eta}_{G}^{2}\] = .206, a main effect of *instructions, F*(1,98) = 2.69, *p* = .104, \[
\hat{\eta}_{G}^{2}\] = .027, and no interaction, *F*(1,98) = 0.31, *p* = .580, \[
\hat{\eta}_{G}^{2}\] = .003. We also tested forced-choice performance against chance baseline (1/5 = .2) using planned contrasts. Performance in the probabilistic/sequence-concealed condition did not differ from chance, *M* = 0.30, *t*(98) = 1.51, *p* = .067. (with a small number of participants showing above-chance performance). Participants in the probabilistic/sequence-revealed condition performed above chance, *M* = 0.38, *t*(98) = 2.58, *p* = .006. With mixed-deterministic materials, both groups of participants clearly performed above chance (*M* = 0.62, *t*(98) = 5.67, *p* < .001 in the sequence-concealed condition, and *M* = 0.77, *t*(98) = 8.25, *p* < .001 in the sequence-revealed condition).

Participants who worked on mixed-deterministic materials were well able to acquire substantial amounts of explicit sequence knowledge that they were also able to verbally report in our post-experimental interview. Participants who had received advance knowledge about the sequence performed better than those who had not. In contrast, most participants who worked on probabilistic materials were not able to acquire substantial amounts of such knowledge. Those who had received advance knowledge about the sequence could report above-chance knowledge on the forced-choice questions, but the amount of knowledge was small (i.e., much smaller than that obtained by participants in the mixed-deterministic/sequence-concealed group). Most of these participants appear to have forgotten the structure of the sequence, perhaps because they were not able to effectively use this knowledge while performing the SRTT. A complete overview of results from the post-experimental interview can be found in Appendix B.

### Diffusion model analyses

In a next step, we applied the diffusion model to the present data. We first report parameter estimates for the whole sample of participants. In a second step, we contrast the parameters involved in the expression of implicit knowledge from those reflecting the expression of explicit knowledge in a plan-based fashion. To this end, we report modelling results for a subset of participants who (1) worked on probabilistic materials, were not revealed any sequence knowledge, and, according to the post-experimental interview, remained fully implicit about the sequence, and (2) for a subset of participants who worked on mixed-deterministic materials, were instructed about the sequence, and reported comprehensive explicit sequence knowledge.

#### Whole sample

**Figure 6:**
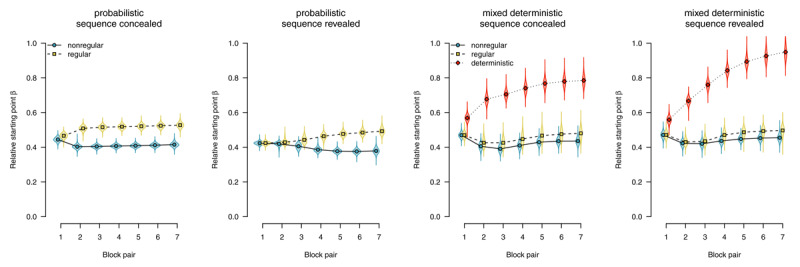
Starting point of the diffusion process. Points represent posterior means of the group-level parameters \[
\mu _{kb}^{\left(\beta \right)}\], violins represent the corresponding posterior densities.

**Figure 7:**
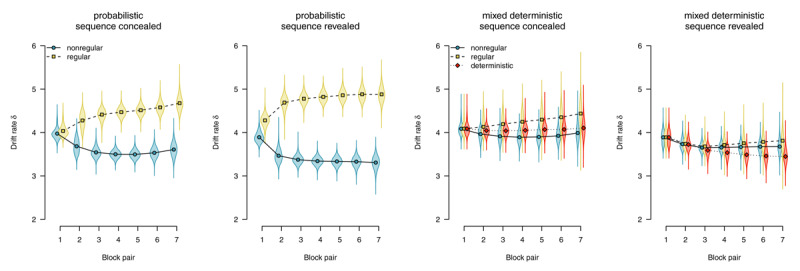
Average evidence accumulation (drift rate). Points represent posterior means of the group-level parameters \[
\mu _{kb}^{\left(\delta \right)}\], violins represent the corresponding posterior densities.

**Figure 8:**
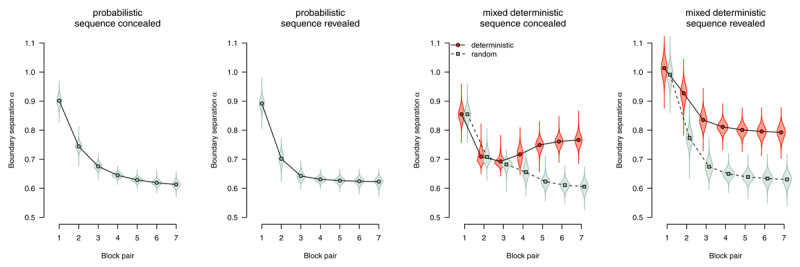
Boundary separation (response caution). Points represent posterior means of the group-level parameters \[
\mu _{{{k}^{{\rm *}}}b}^{\left(\alpha \right)}\], violins represent the corresponding posterior densities.

**Figure 9:**
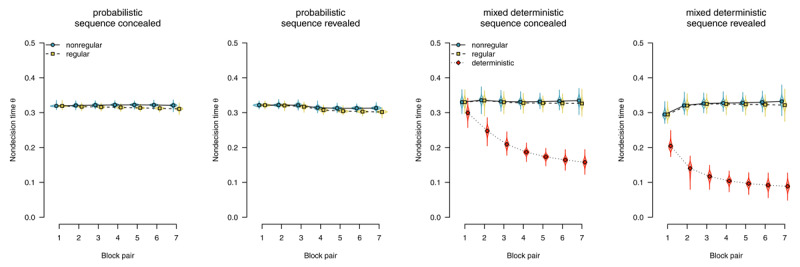
Nondecision times, separated by stimulus-location regularity. Points represent posterior means of the group-level parameters \[
\mu _{kb}^{\left(\theta \right)}\], violins represent the corresponding posterior densities.

**Figure 10:**
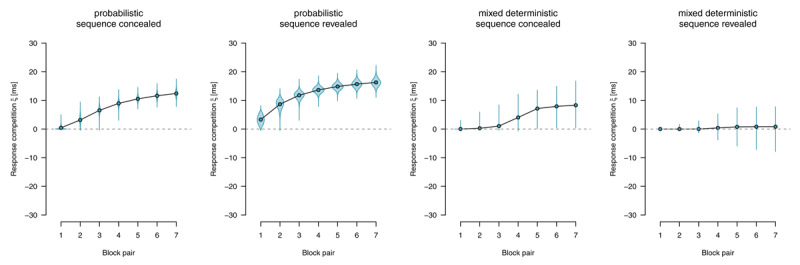
Response-competition parameter ξ, capturing differences in nondecision time between motor-regular and motor-nonregular responses. Positive values imply faster responses for motor-regular responses. Points represent posterior means of the group-level parameters \[
\mu _{b}^{\left(\xi \right)}\], violins represent the corresponding posterior densities.

Parameter estimates are depicted in Figures 6,7,8,9,10. We report Bayes Factors (*BF*) for model comparisons that tested the effects of experimental manipulations on model parameters.

##### Starting point

The *starting point* parameter captures information that is available *before* the evidence accumulation process starts (i.e., before the imperative stimulus is presented). In the present application of the diffusion model, it could be biased either towards regular (i.e., upper boundary) or towards nonregular responses (i.e., lower boundary). Figure 6 shows parameter estimates of the starting point. Participants who worked on probabilistic materials were increasingly biased to select regular responses: In both the sequence-concealed condition (*BF*_10_ > 1,000) and the sequence-revealed condition (*BF*_10_ > 1,000) the *BF* clearly favored the alternative hypothesis of an effect of regularity. Participants who worked on mixed-deterministic materials showed a strong bias towards the regular response in deterministic compared with random blocks: In both the sequence-concealed condition (*BF*_10_ > 1,000) and the sequence-revealed condition (*BF*_10_ > 1,000), Bayes Factors clearly favored the alternative hypothesis of greater starting point estimates in deterministic (vs. random) blocks. Comparing regular and nonregular trials in random blocks, we found evidence against an effect of regularity (sequence concealed: *BF*_01_ = 7.61, sequence revealed: *BF*_01_ = 5.59).

##### Drift rate

The drift rate reflects the speed of evidence accumulation (i.e., information uptake from the stimulus and/or a match with memory). Figure 7 shows drift rate estimates. With probabilistic materials, the expression of sequence learning is mediated by faster evidence accumulation for regular (compared with nonregular) trials. In the sequence-concealed condition, we found *BF*_10_ > 1,000, in the sequence-revealed condition, we found *BF*_10_ > 1,000. By contrast, with deterministic materials, we consistently found evidence against an effect on drift rate. Comparing deterministic with random blocks, we found evidence against an effect in both the sequence-concealed (*BF*_01_ = 18.45) and the sequence-revealed condition (*BF*_01_ = 6.03). Comparing regular and nonregular trials in random blocks also revealed evidence against an effect of regularity (sequence concealed: *BF*_01_ = 2.82, sequence revealed: *BF*_01_ = 9.06).

##### Boundary separation

The boundary separation parameter reflects response caution: Higher values reflect more separated boundaries, hence more cautious responding. Figure 8 shows boundary separation parameter estimates. In all conditions, we find strong evidence for an overall decrease in response caution (all *BF*_10_ > 1,000), Participants responded more liberally over time to accommodate the time pressure in this experiment. However, in deterministic blocks, participants were able to exploit the speedup attributable to changes in other model parameters (starting point and nondecision time) to maintain higher response caution (sequence concealed: *BF*_10_ = 375.10, sequence revealed: *BF*_10_ > 1,000).

##### Nondecision time

In the diffusion model, the *nondecision time* parameter subsumes all processes (i.e., perceptual and motor) occurring before and after the response-selection process.

Figure 9 shows nondecision times as a function of stimulus regularity. With probabilistic materials, we found evidence for the absence of sequence-specific effects, with *BF*_01_ = 3.23 in the sequence-concealed condition, and *BF*_01_ = 2.62 in the sequence-revealed condition. We therefore conclude that, if participants performed the SRTT in a stimulus-based fashion, it was neither stimulus detection nor encoding that mediated the expression of sequence learning.

A similar pattern could be observed in participants who worked on mixed-deterministic materials: When considering random blocks, we found evidence for an absence of a difference in nondecision time attributable to sequence learning, with *BF*_01_ = 38.83 in the sequence-concealed condition, and *BF*_01_ = 33.94 in the sequence-revealed condition. Comparing deterministic with random blocks, by contrast, we found strong evidence for a rapid decrease in nondecision time for deterministic materials, with *BF*_10_ > 1,000 in the sequence-concealed condition, and *BF*_10_ > 1,000 in the sequence-revealed condition. We interpret this finding as indicative that a switch to plan-based action control allowed participants to largely skip the process of stimulus detection and encoding; instead, during the RSI, they already prepared a response that was executed as soon as something appeared on the screen.

##### Response competition

We applied an extended diffusion model with an additional *response-competition* parameter reflecting differences in nondecision times between motor-regular and motor-nonregular responses. Figure 10 shows response-competition parameter estimates. For probabilistic materials, we found clear evidence for a response competition effect, (sequence concealed: *BF*_10_ > 1,000, sequence revealed *BF*_10_ > 1,000). For deterministic materials, we found such an effect if the sequence was concealed, *BF*_10_ = 869.53; but found evidence against such an effect if the sequence was revealed, *BF*_01_ = 48.10.

#### Subset analyses

To more clearly pin down the expression of implicit versus explicit sequence knowledge, using the post-experimental-interview data, we classified participants (within each condition) into three groups: (1) Participants who indicated that they probably were in a random condition and only reproduced less than two transitions correctly in both the forced-choice and free-recall test (henceforth coined *implicit* groups), (2) participants who indicated that they probably were in a sequenced condition, and reproduced six transitions correctly in both free-recall and forced-choice test (the *explicit* group), and (3) participants who fell in-between these criteria (the *intermediate* groups). We then re-estimated the DDM for these sub-groups if there were at least four participants in the respective group, and compared parameter estimates across groups.

Results showed that, in general, the overall patterns of parameter estimates were not affected by the amount of explicit knowledge: Parameter patterns were largely identical across all participants in the probabilistic groups (regardless of whether they had received advance knowledge about the sequence, or had acquired some explicit knowledge during their time on the task). Similarly, parameter patterns were also largely identical among the subgroups working on mixed-deterministic material, regardless of the source or amount of explicit knowledge.^2^ These findings demonstrate that the presence of explicit knowledge *per se* does not necessarily affect performance. Participants also need the opportunity, afforded by the deterministic blocks, to express that knowledge in a plan-based manner.

**Figure 11:**
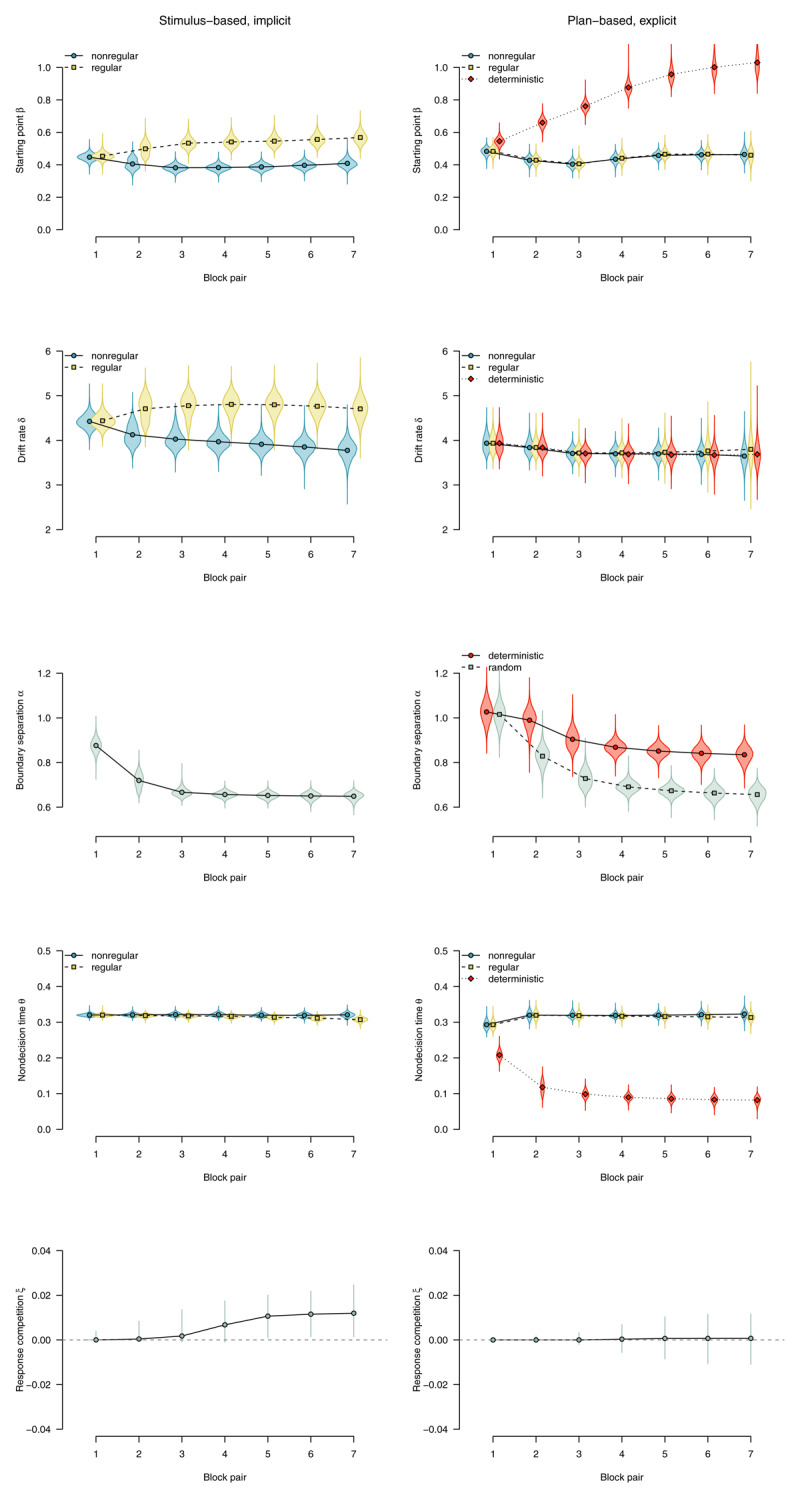
DDM parameters for stimulus-based (on the left) versus plan-based SRTT performance (on the right). Points represent posterior means of the group-level parameters, violins represent the corresponding posterior densities.

To characterize the expression of implicit sequence knowledge, we used the parameter estimates from the group that worked on probabilistic materials in the sequence concealed condition and did not show any indication of explicit knowledge (i.e., the *implicit* subgroup). These participants could only perform the task in a stimulus-based manner. The left panels of Figure 11 show parameter estimates for this subgroup. In line with our whole-sample results, if the SRTT was performed in a stimulus-based fashion and participants remained implicit about the sequence, such implicit learning affected the response-selection process, with a tendency to select the rule-adhering response (starting point β), and faster evidence accumulation for rule-adhering stimuli (drift rate δ). We found no effect of stimulus regularity on nondecision time θ, but a robust effect of motor regularity (response competition ξ).

To characterize the expression of explicit knowledge, we used the parameter estimates from the group of participants who worked on mixed-deterministic materials, received full advance sequence knowledge, and were able to report the complete sequence at the end of the experiment. These participants most likely performed the task in a plan-based fashion. The right panels of Figure 11 show parameter estimates for this subgroup. Plan-based action control in deterministic blocks was reflected in an anticipation of the next response (starting point β), indicating that response selection is almost completed when the stimulus appears on the screen. Evidence accumulation from the stimulus (drift rate δ) was not affected by a switch to plan-based action control, indicating that in such a processing mode, the little information that was necessary to select the response (i.e., the appearance of a stimulus on the screen) was processed as efficiently as in random blocks. Nondecision time θ was dramatically reduced in deterministic blocks (compared with random blocks), indicating that stimulus detection and/or encoding could be bypassed. Participants were also able to use their performance advantage to respond more cautiously in deterministic versus random blocks (boundary separation α). There was no effect of motor regularity on nondecision time (i.e., no response-competition effect).

To summarize, implicit knowledge affected the drift rate as well as the starting point of the diffusion process, and also caused response competition. In contrast, expressing explicit knowledge in a plan-based fashion did not affect the drift rate; it was most prominently reflected in a decrease of nondecision time and an effect on the starting point. While the starting point was affected in both cases, implicit sequence knowledge selectively involved an effect on drift rate, and plan-based expression of explicit knowledge selectively affected overall nondecision time.

## Discussion

We set out to pinpoint the cognitive processes that are involved in the expression of implicit versus explicit sequence knowledge. We found that implicit sequence knowledge gradually affects cognitive processes during stimulus-based task processing, whereas explicit knowledge supports a switch to plan-based processing.

In our baseline condition (probabilistic materials, sequence concealed), we found that sequence learning was expressed by a combination of effects on starting point, drift rate, and response-competition bias. Over the course of training, the starting point was shifted towards the regular response, indicating that even before the imperative stimulus was presented, the response-selection process was increasingly biased towards the rule-adhering response option. Moreover, as suggested by higher drift rates for regular trials, evidence accumulation was faster for rule-adhering trials, indicating that either the information from the imperative stimulus or the stimulus-response rule was processed more efficiently. Nondecision time was invariant to stimulus-location regularity, but varied by response-location regularity (i.e., we found an effect of response competition), indicating an additional response-based learning effect that might be located at the motor level. To summarize, response selection appears to be involved in implicit sequence learning. This is in line with previous findings supporting S–R representations (Schwarb & Schumacher, 2012), and suggests the involvement of a multidimensional system (i.e., combining stimulus and response dimensions) or a unidimensional learning system where spatial features of stimulus and response are jointly represented (Haider et al., 2020). In addition, we found evidence for response-competition bias toward the regular responses, suggesting the involvement of motor facilitation. As the present study confounded effectors with response locations, it could be explained by both effector-specific or general effects (Verwey & Clegg, 2005; Willingham, Wells, Farrell, & Stemwedel, 2000). Future research is needed to separate these two factors and their effects on the response-competition parameter.

Participants who worked on probabilistic materials and received advance knowledge of the sequential structure, and were encouraged to use this knowledge, were hard-pressed to express this extra knowledge. Overall, the performance pattern of this group was highly similar to that of the sequence-concealed group, with sequence learning being reflected in effects on starting point, drift rate, and response competition. Compared with the sequence-concealed condition, the effect on drift rate was increased, whereas the effect on starting point was reduced. Future research is needed to determine whether these differences are robust and reflect participants’ attempts to express their explicit knowledge.

By contrast, participants who worked on mixed-deterministic materials showed a qualitatively distinct pattern of parameter estimates. It was characterized by a much stronger effect on the starting point and a steep reduction in nondecision time. In addition, boundary separation was increased in the deterministic blocks. There were no effects on drift rate or the response-competition parameter. This parameter pattern suggests that participants engaged in plan-based task performance, using their explicit knowledge to anticipate the stimulus and prepare their response. This anticipation was so successful (i.e., the starting point was so strongly shifted towards the *regular* boundary) that participants needed to extract only very little information from the stimulus during the decision process. (The present finding of no effect on drift rate in the deterministic condition should therefore be interpreted with caution: It may simply reflect the fact that, in our study, virtually no information had to be sampled from the stimulus.) Because plan-based performance speeded up participants’ responses in the deterministic blocks, they could afford to increase their response caution (i.e., boundary separation) in these blocks.

Participants who worked on mixed-deterministic materials but did not receive advance sequence knowledge showed a similar pattern, with smaller effects overall. In this condition, a substantial subset of participants have apparently acquired explicit sequence knowledge and switched to a plan-based processing mode. The only difference was the (small) effect on the response-competition parameter, which was found in the mixed-deterministic/sequence-concealed group but not in the sequence-revealed group. Because the proportion of regular motor key presses is comparable between all four conditions, the absence of a response-competition effect for the mixed-deterministic/sequence-revealed group suggests that this parameter does not capture a purely motor-learning effect that is a mere by-product of repeatedly performing the same sequence of motor key presses. Instead, it might depend on performing the task in a stimulus-driven manner. Because participants who worked on mixed-deterministic materials in the sequence-revealed condition were able to work in a plan-based fashion from the very start, they might not have engaged in a sufficient amount of stimulus-based task performance in order to acquire a response-competition effect. In contrast, participants who worked on mixed-deterministic materials in the sequence-concealed condition were not able to engage in plan-based task performance from the start. The response-competition effect may be explained by the notion that this learning effect develops if and only if participants perform the task in a stimulus-based fashion. Clearly, future empirical work is needed to clarify the interpretation of this effect.

### Limitations

It may be argued that our manipulation of sequence knowledge was not successful, given that revealed sequence knowledge was largely forgotten with probabilistic materials. However, we found a clear effect of instructions on error rates for nonregular trials, indicating that at least some participants tried to implement their sequence knowledge while performing the task.

Instead of reflecting the absence of implicit learning effects on stimulus detection or encoding, the lack of an effect on nondecision time may point to the fact that both stimulus detection and encoding were relatively easy in our experiment (i.e., that we observed a floor effect). Alternatively, such a learning effect may truly be nonexistent. Earlier findings of purely observational (Howard, Mutter, & Howard, 1992; Song, Howard, & Howard, 2008) or parallel learning of stimulus locations (Mayr, 1996) may then reflect either an artifact of explicit knowledge or implicit learning of spatial locations in a unidimensional system (c.f., Eberhardt et al., 2017) that is expressed at the response-selection stage.

The DDM has been widely successful in capturing and explaining performance in many speeded-choice tasks, and we therefore deem it an excellent starting point for modelling performance in the SRTT. However, it makes a set of processing assumptions that might be violated in a specific application. Most importantly, it assumes that stimulus encoding, response selection, and response execution are organized sequentially, an assumption that might be violated in sequence learning. This is particularly relevant because parallel architectures have been proposed for motor-behavior tasks such as the SRTT (e.g., Logan, 1988; Verwey, Shea, & Wright, 2015). Hence, unless the model receives further validation for SRT tasks, the present set of findings may be alternatively accounted for by theories involving parallel processes.

As another point of criticism, it may be argued that the DDM, as a decision model, may be less useful for conditions involving plan-based actions to the degree that plan-based action control requires little information from the stimulus to arrive at a decision. Note, however, that the DDM was developed for decisions from memory, and the use of explicit knowledge may well be characterized as involving such memory-based decisions (i.e., recalling the next element of the sequence).

### Future Directions

Extensions of the SRTT allow to orthogonally manipulate stimulus vs. motor sequences by changing the stimulus-response mapping on each trial (e.g., Eberhardt et al., 2017; Goschke & Bolte, 2012) Such extended designs provide the opportunity to validate the DDM’s application to the SRTT, and to further pin down the interpretation of the observed parameter changes: To clarify how to interpret the response-competition effect that we found for stimulus-based implicit learners, it could be tested whether motor sequences, but not stimulus sequences selectively influence this parameter. Moreover, the effector-specificity of this effect could be investigated using a transfer task where effectors are changed but response locations are kept constant (c.f., Verwey & Clegg, 2005). If sequence learning of stimulus locations guides spatial attention on the screen (as suggested by Mayr, 1996), it should selectively influence stimulus detection (i.e., nondecision time). In the present work, we did not find such an effect, but stimulus detection was very easy; increasing the difficulty of stimulus detection (e.g., by presenting irrelevant stimuli in the other locations) might give rise to an effect of sequence learning on stimulus detection when responses are unrelated to stimulus locations.

It is an unresolved issue whether two distinct learning systems (one multidimensional, one unidimensional) are necessary to explain the pattern of findings in the sequence-learning literature (Barth, Stahl, & Haider, 2023), one of the reasons being that it is not clear along which dimensions information is considered to be processed separately within the unidimensional system. Our findings indicate that response selection is the locus of sequence learning in the SRTT, and that, therefore, only joint representations of stimulus and response features may subserve the standard SRTT effect. A unidimensional learning system that is separated along the lines of stimulus and response can, therefore, not explain our findings. However, if the unidimensional system is separated along abstract features such as location (as suggested by Eberhardt et al., 2017; Haider et al., 2020), a unidimensional learning system could indeed provide the information necessary to guide spatial response selection. With the diffusion model introduced here, it would be possible to more directly test the separation of information processing that is implied by modularization. For instance, if other stimulus features not only affect stimulus processing (i.e., nondecision time) but also response-selection parameters, this would clearly speak against such modularized processing (Barth et al., 2023).

In the present work, we used a regression approach at the group-level means to model the changes in parameter estimates over the course of learning. Most recently, considerable efforts have been made to model both gradual and sudden shifts in parameter estimates (Gunawan, Hawkins, Kohn, Tran, & Brown, 2022; L. Schumacher et al., 2024). Such non-stationary models may be used to model individual learning curves at the level of a single transition, providing a principled on-line measure of a switch to plan-based action control in the SRTT. Moreover, these methods allow to more closely investigate the temporal dynamics of both implicit and explicit sequence learning (c.f., Musfeld, Souza, & Oberauer, 2023). For a regression-based approach, recent advances in Bayesian estimation carry the potential to facilitate such endeavors: Henrich, Hartmann, Pratz, Voss, and Klauer (2024) implemented the seven-parameter DDM in Stan (Carpenter et al., 2017), a probabilistic programming language that uses Hamiltonian Monte Carlo, a sampling algorithm that provides better computational efficiency than the sampling algorithms used in the present work (Betancourt, 2017).

## Conclusion

The present study is the first to use the diffusion model to analyze processes underlying performance in the SRTT. In this first step, we focused on the effects of implicit learning and plan-based expression of explicit knowledge. Our results demonstrate the usefulness of the diffusion model in research on implicit learning. They indicate that implicit sequence learning in the SRTT guides response selection, supporting either multidimensional accounts of implicit sequence learning (E. H. Schumacher & Hazeltine, 2016) or, alternatively, unidimensional accounts that assume that features from the same abstract dimension (e.g., spatial location) of both stimulus and response are jointly represented (Haider et al., 2020).

In addition, we find evidence for response competition that is independent of response selection. More research is needed to investigate whether this effect represents effector-specific motor learning (Verwey & Clegg, 2005) or is linked to abstract response categories (Willingham et al., 2000). Supporting the recent tests of the Unexpected Event Hypothesis (Lustig et al., 2022), strong decreases in response time are readily accounted for as a consequence of a switch to plan-based action control. This switch is possible only after the sequence has become explicit, and only if the structure of the task makes such a change beneficial for performance. When implemented, such a strategy shift results in an anticipation of the next response even before the imperative stimulus is presented, allowing participants to largely bypass stimulus processing (as reflected in reduced interference effects in Stroop and Simon tasks, Haider et al., 2011; Koch, 2007).

## Figures and Tables

**Figure A1:**
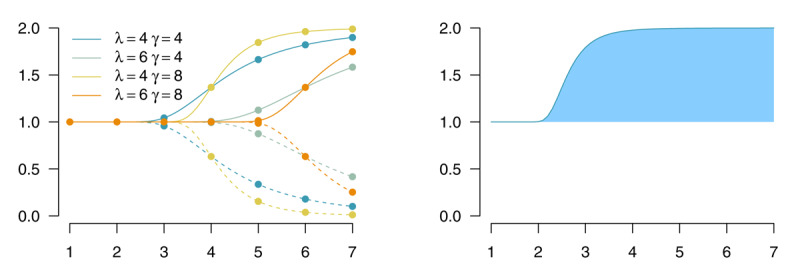
Left panel: Shifted and stretched exponential functions for different values of λ (location) and γ (shape), with υ = 1 and ι ∈ {0,2}. Right panel: The average effect ψ corresponds to the area under the curve (blue) between initial limit and the function’s value *f*(*b*).

## Data Availability

This manuscript was written in R Markdown with the R package papaja (Aust & Barth, 2024). Data, code and materials necessary to reproduce the analyses reported in this article are available at https://github.com/methexp/cpl-public.

## Footnotes

We used R (Version 4.4.3; R Core Team, 2025) and the R packages *afex* (Version 1.4.1; Singmann, Bolker, Westfall, Aust, & Ben-Shachar, 2025), *coda* (Version 0.19.4.1; Plummer, Best, Cowles, & Vines, 2006), *cplmodels* (Version 0.1.0; Barth, 2025a), *emmeans* (Version 1.11.0; Lenth, 2025), *logspline* (Version 2.1.22; Kooperberg, 2024), *papaja* (Version 0.1.3; Aust & Barth, 2024), *rtdists* (Version 0.11.5; Singmann, Brown, Gretton, & Heathcote, 2022), *runjags* (Version 2.2.2.4; Denwood, 2016), and *tinylabels* (Version 0.2.5; Barth, 2025b) for all our analyses.

Participants who worked on mixed-deterministic materials and received advance knowledge of the sequence but were, according to our criteria, classified as being implicit about the sequence also showed an above-zero response-competition effect. Evidently, these participants failed to remember the sequence or did not read the instructions properly and, therefore, also engaged in stimulus-based processing of the SRTT.

## Appendix A

### Model specification

We implemented the drift-diffusion model in JAGS (Plummer, 2003) and fit the model to each between-subjects condition, separately. Response times and accuracy on each trial *n* were modeled as coming from a Wiener distribution with the models’ *core* parameters boundary separation \[
{{\alpha}_{i{{k}^{{\rm *}}}b}}\], starting point β*_n_*, drift rate δ*_n_* and nondecision time τ*_n_*

\[
{{y}_{n}}\sim\mathcal{W}\ \left({{\alpha}_{i{{k}^{{\rm *}}}b}},\ {{\beta}_{n}},\ {{\delta}_{n}},\ {{\tau}_{n}} \right)\]

Boundary separation is assumed to vary by participant *i* and block pair *b*. For mixed-deterministic materials, it also varies by condition *k**, denoting deterministic vs. random blocks.

\[
{{\alpha}_{i{{k}^{{\rm *}}}b}}=\zeta _{i{{k}^{{\rm *}}}b}^{\left(\alpha \right)}\]

The other core parameters are assumed to vary by trial, and are assumed to come from truncated normal distributions with standard deviations *s_i_*.

\[
\beta_{n}\sim {\rm N}_{.01}^{.99} \left(\zeta_{ikb}^{(\beta)} + p^{(\beta)}\kappa_{ij}^{(\beta)}, s_{i}^{(\beta)} \right)
\]

\[
{{\delta}_{n}}\sim{\rm N}\left(\zeta _{ikb}^{\left(\delta \right)}\ +\ {{p}^{\left(\delta \right)}}\kappa _{ij}^{\left(\delta \right)},\ s_{i}^{\left(\delta \right)} \right)\]

\[
{{\tau}_{n}}\sim{{{\rm N}}_{.001}}\left(\zeta _{ikb}^{\left(\theta \right)}+{{p}^{\left(\theta \right)}}\kappa _{ij}^{\left(\theta \right)}+\zeta _{il}^{\left(\xi \right)}+{{p}^{\left(\xi \right)}}\kappa _{il}^{\left(\xi \right)},s_{i}^{\left(\tau \right)} \right)
\]

where \[
\zeta _{ikb}^{(s)}\] is an individual’s condition (nonregular, regular, deterministic) mean in block pair *b, p* is a scaling factor, and κ*_ij_* represent an individual’s stimulus-location-dependent deviations from the individual’s condition mean.

Participant-level parameters \[
\zeta _{ikb}^{(s)}\] are assumed to be normally distributed,

\[
\zeta _{ikb}^{(s)}\sim{\rm N}\left(\mu _{kb}^{(s)},\ {{\sigma}^{(s)}} \right)\]

where

\[
{{\sigma}^{(s)}}\sim{{q}^{(s)}}t_{df=I-1}^{+}
\]

and *q*^(*s*)^ is a scaling factor, and \[
t_{df}^{+}
\] is a half-*t* distribution with *I*–1 degrees of freedom restricted to positive values.

In the SRTT, response times and accuracy vary substantially by stimulus and/or response location. Therefore, for each participant *i* and stimulus location *j* = 1, …, 6, we included normally distributed parameters κ*_ij_* to capture such differences

\[
\kappa _{ij}^{(s)}\sim{\rm N}\left(\mu _{j}^{{{\kappa}^{(s)}}},\sigma _{j}^{{{\kappa}^{(s)}}} \right)\ {\rm for}\ j=1,\ \ldots,\ 5\]

and

\[
\kappa _{i6}^{(s)}={-}\underset{j=1}{\overset{5}{\sum}}\,\kappa _{ij}^{(s)}\]

The means of these parameters are modeled as being normally distributed, where

\[
\mu _{j}^{{{\kappa}^{(s)}}}\sim{\rm N}\left(0,\ 1 \right)\,{\rm for} ~ j=1,\ \ldots,\ 5\]

and

\[
\mu _{6}^{{{\kappa}^{(s)}}}=-\underset{j=1}{\overset{5}{\sum}}\,\mu _{j}^{{{\kappa}^{(s)}}}\]

Standard deviations were modeled as coming from (positive) half-*t* distributions

\[
\sigma _{j}^{{{\kappa}^{(s)}}}\sim {t^{+}_{df=I-1}}
\]

The condition means are defined as the weighted sum of one or more shifted and stretched exponential functions *f*(*b*),

\[
{{\mu}_{kb}}=\underset{m=1}{\overset{3}{\sum}}\,{{w}_{km}}{{f}_{m}}{{\left(b \right)}_{{{\lambda}_{m}},{{\gamma}_{m}},{{\upsilon}_{m}},{{\iota}_{m}}}}\]

We use these functions in a similar fashion as model terms in linear models: Condition means are the weighted sum of multiple temporally-changing functions (instead of regression coefficients), and the condition weights *w_km_* chosen in a way that the first function is equivalent to an intercept term, the second function is equivalent to the effect of regular vs. nonregular trials, and the third function is equivalent to the effect of deterministic trials vs. the average of regular and nonregular trials; amounting to a Helmert coding scheme.

The functions (depicted in Figure A1) are defined as

\[
{{f}_{m}}\left(b \right)={{\upsilon}_{m}}+\left({{\iota}_{m}}-{{\upsilon}_{m}} \right){{e}^{-{{\left(\frac{b}{{{\lambda}_{m}}} \right)}^{-{{\gamma}_{m}}}}}}\]

where υ*_m_* is the initial limit, ι*_m_* is the final limit. λ*_m_* is the temporal location of the inflection point of this function, and γ*_m_* is a stretching exponent (shifting the function’s steepness).

The final limit ι*_m_* may be re-written as

\[
{{\iota}_{m}}=\frac{{{\gamma}_{m}}{{\psi}_{m}}B}{{{\lambda}_{m}}\Gamma \left(\gamma _{m}^{-1} \right)}+{{\upsilon}_{m}}\]

where *B* is the number of block pairs, Γ(.) is the Gamma function, and ψ*_m_* is the *average effect*, i.e., the average difference between *f_m_*(*b*) and the initial limit υ*_m_*.

We use these average effects ψ*_m_* as the target of inference to test for overall changes (first function), the effect of nonregular vs. regular trials (second function), and the effect of deterministic vs. random blocks (third function). Bayes Factors (BF) are calculated using the Savage-Dickey density ratio (Wagenmakers, Lodewyckx, Kuriyal, & Grasman, 2010) from logspline densities (Kooperberg & Stone, 1992).

## Appendix B

### Direct Measures of Explicit Sequence Knowledge

**Table B1:** Number of correctly reproduced transitions in free-recall and forced-choice tests. Numbers for only those participants who indicated that they were in a sequenced condition are given in parentheses.

FREE RECALL	FORCED CHOICE						

	0	1	2	3	4	5	6

probabilistic, sequence concealed							

0	8 (1)	1 (1)	2 (2)	.	.	.	1 (0)

1	1 (1)	2 (0)	2 (2)	2 (1)	.	.	.

2	1 (1)	.	.	.	.	.	.

3	.	1 (1)	1 (1)	.	.	.	.

4	.	1 (1)	.	.	.	1 (1)	.

6	.	.	.	.	.	1 (0)	2 (1)

mixed deterministic, sequence concealed							

0	.	1 (1)	1 (0)	1 (0)	.	.	.

1	1 (1)	.	2 (2)	.	.	.	.

2	.	1 (1)	3 (2)	1 (1)	.	.	.

3	.	.	.	.	.	.	.

4	.	.	.	.	.	.	.

6	.	.	.	1 (1)	1 (1)	2 (2)	8 (8)

probabilistic, sequence revealed							

0	7 (1)	.	1 (1)	.	.	.	.

1	.	2 (2)	2 (1)	.	1 (0)	.	.

2	.	.	.	.	.	.	.

3	2 (0)	.	.	2 (2)	1 (0)	1 (1)	.

4	.	.	1 (0)	.	.	.	.

6	.	.	.	1 (1)	1 (1)	1 (0)	3 (2)

mixed deterministic, sequence revealed							

0	.	1 (1)	.	.	.	.	.

1	.	2 (1)	.	.	.	.	.

2	.	.	.	.	.	.	.

3	1 (1)	1 (1)	.	.	1 (1)	.	1 (1)

4	.	.	.	.	.	.	1 (1)

6	.	.	1 (1)	.	2 (2)	.	15 (15)
